# Editorial: Point-of-care diagnostics for veterinary use

**DOI:** 10.3389/fvets.2023.1206117

**Published:** 2023-05-11

**Authors:** Yogesh Chander

**Affiliations:** Varigen Biosciences Corporation, Madison, WI, United States

**Keywords:** point-of-care (POC), isothermal (DNA) amplification, CRISPR, olfaction, dried blood sample (DBS)

This Research Topic focused on advances in development of point-of care (POC) diagnostics for veterinary use. Academic researchers from across the world have come together to develop novel approaches for pathogen detection. Four articles (one review and three original research papers) of current importance were published in this Research Topic.

As the world's population grows, tremendous pressure is placed on aquaculture and agriculture to supply adequate food. This requires an increase in production and food safety to meet the needs and preferences of different populations. Food safety implies keeping the supply chain free of food-borne pathogens and keeping food animals (livestock and aquaculture) and plants free of pathogens. New technologies are required for prompt and accurate detection of pathogens as many diseases are known to cause significant economic losses to producers. Besides this, many domestic and wild animals are known act as a reservoir for zoonotic diseases thus, posing a significant risk to human populations.

During the last couple of decades many technological advances have been made to develop and improve diagnostics for infectious diseases in animals. However, much of the testing is still done in centralized laboratories. During the last few years, POC testing has gained popularity for use in veterinary diagnostics because of its advantages such as (1) simple and easy to use, (2) rapid turnaround; and (3) no need to ship samples to central lab. Velayudhan and Naikare presents an overview of different types of POC tests currently available for companion and food animal disease diagnostics, tests in the pipeline and their advantages and disadvantages.

Topics touch upon use of novel techniques to develop next generation of diagnostic assays. Huang et al. report on the development of a sensitive and visual assay for rapid detection of feline calicivirus based on isothermal amplification assay (recombinase polymerase amplification) and CRISPR-Cas13a trans-cleavage activity. Sensitivity of this assay was 5.5 copies/μL with a positivity rate of 67.9% (38/56) as compared to that of RT-qPCR (44.6%, 25/56) using clinical samples. To develop a highly sensitive and specific diagnostic test it is important to have sufficient number of relevant and high-quality samples for validation studies. Blood and urine are common biological samples for routine clinical analysis and identification of biomarkers. Allaway et al. reported on the suitability of dried blood spots for biomarker analysis. This method allows use of minimal blood volume and faster processing of samples as well as relatively low cost for sampling/storage. Juge et al. reported on the use of dogs for early detection of bovine respiratory disease, a difficult to diagnose condition in cattle.

This Research Topic highlights the advances made in the field of POC testing for veterinary diagnostics. Even though there are only four articles in this Research Topic, continuous efforts are being made around the globe too develop better diagnostics to keep animals safe from diseases and be prepared to meet any challenge.

## Author contributions

YC contributed to the editorial and to the management of the whole Research Topic.

